# Multidisciplinary recommendations for the management of CAR-T recipients in the post-COVID-19 pandemic era

**DOI:** 10.1186/s40164-023-00426-x

**Published:** 2023-07-27

**Authors:** Tingting Zhang, Weiwei Tian, Shuang Wei, Xinyi Lu, Jing An, Shaolong He, Jie Zhao, Zhilin Gao, Li Li, Ke Lian, Qiang Zhou, Huilai Zhang, Liang Wang, Liping Su, Huicong Kang, Ting Niu, Ailin Zhao, Jing Pan, Qingqing Cai, Zhenshu Xu, Wenming Chen, Hongmei Jing, Peng Li, Wanhong Zhao, Yang Cao, Jianqing Mi, Tao Chen, Yuan Chen, Ping Zou, Veronika Lukacs-Kornek, Christian Kurts, Jian Li, Xiansheng Liu, Qi Mei, Yicheng Zhang, Jia Wei

**Affiliations:** 1 Cancer Center, Shanxi Bethune Hospital, Shanxi Academy of Medical Sciences, Tongji Shanxi Hospital, Third Hospital of Shanxi Medical University, Taiyuan, 030032 Shanxi China; 2 Department of Hematology, Tongji Shanxi Hospital, Shanxi Bethune Hospital, Shanxi Academy of Medical Sciences, Third Hospital of Shanxi Medical University, Taiyuan, 030032 Shanxi China; 3 Department of Respiratory and Critical Care Medicine, Tongji Hospital, Tongji Medical College, Huazhong University of Science and Technology, Wuhan, 430030 Hubei China; 4 Department of Respiratory and Critical Care Medicine, Tongji Shanxi Hospital, Shanxi Bethune Hospital, Shanxi Academy of Medical Sciences, Third Hospital of Shanxi Medical University, Taiyuan, 030032 Shanxi China; 5 Sino-German Joint Oncological Research Laboratory, Shanxi Bethune Hospital, Shanxi Academy of Medical Sciences, Taiyuan, 030032 Shanxi China; 6 School of Public Health, Shanxi Medical University, Taiyuan, 030000 Shanxi China; 7 Division of Cardiology, Department of Internal Medicine, Tongji Hospital, Tongji Medical College, Huazhong University of Science and Technology, Wuhan, 430030 Hubei China; 8 Department of Cardiovascular Medicine, Tongji Shanxi Hospital, Shanxi Bethune Hospital, Shanxi Academy of Medical Sciences, Third Hospital of Shanxi Medical University, Taiyuan, 030032 Shanxi China; 9 Department of Lymphoma, Tianjin Medical University Cancer Institute and Hospital, National Clinical Research Center for Cancer, Tianjin, 300060 China; 10 Department of Hematology, Beijing TongRen Hospital, Capital Medical University, Beijing, 100730 China; 11 Shanxi Province Cancer Hospital, Shanxi Hospital Affiliated to Cancer Hospital, Chinese Academy of Medical Sciences, Cancer Hospital Affiliated to Shanxi Medical University, Taiyuan, 030032 Shanxi China; 12 Department of Neurology, Tongji Hospital, Tongji Medical College, Huazhong University of Science and Technology, Wuhan, 430030 Hubei China; 13 Department of Neurology, Tongji Shanxi Hospital, Shanxi Bethune Hospital, Shanxi Academy of Medical Sciences, Third Hospital of Shanxi Medical University, Taiyuan, 030032 Shanxi China; 14 Department of Hematology, West China Hospital, Sichuan University, Chengdu, 610041 Sichuan China; 15 State Key Laboratory of Experimental Hematology, Boren Biotherapy Translational Laboratory, Boren Clinical Translational Center, Beijing GoBroad Boren Hospital, Beijing, 100070 China; 16 Sun Yat-Sen University Cancer Center, Guangzhou, 510060 Guangdong China; 17 Hematology Department, Fujian Medical University Union Hospital, Fujian Institute of Hematology, Fuzhou, 350001 Fujian China; 18 Department of Hematology, Beijing Chaoyang Hospital, Capital Medical University, Beijing, 100020 China; 19 Department of Hematology, Peking University Third Hospital, Beijing, 100191 China; 20 Guangzhou Institutes of Biomedicine and Health, Chinese Academy of Sciences, Guangzhou, 510535 Guangdong China; 21 Department of Hematology, The Second Affiliated Hospital of Xi’an Jiaotong University, Xi’an, 710004 Shanxi China; 22 Department of Hematology, Tongji Hospital, Tongji Medical College, Huazhong University of Science and Technology, Wuhan, 430030 Hubei China; 23 Immunotherapy Research Center for Hematologic Diseases of Hubei Province, Wuhan, 430030 Hubei China; 24 Shanghai Institute of Hematology, Ruijin Hospital Affiliated with Shanghai Jiao Tong University School of Medicine, Shanghai, 200025 China; 25 Department and Institute of Infectious Diseases, Tongji Hospital, Tongji Medical College, Huazhong University of Science and Technology, Wuhan, 430030 Hubei China; 26 Department of Geriatrics, Tongji Hospital, Tongji Medical College, Huazhong University of Science and Technology, Wuhan, 430030 Hubei China; 27 Department of Geriatrics, Tongji Shanxi Hospital, Shanxi Bethune Hospital, Shanxi Academy of Medical Sciences, Third Hospital of Shanxi Medical University, Taiyuan, 030032 Shanxi China; 28 Institute of Hematology, Union Hospital, Tongji Medical College, Huazhong University of Science and Technology, Wuhan, 430022 Hubei China; 29 Institute of Molecular Medicine and Experimental Immunology, University Clinic of Rheinische Friedrich-Wilhelms-University, 53111 Bonn, Germany; 30 Department of Oncology, Tongji Hospital, Tongji Medical College, Huazhong University of Science and Technology, Wuhan, 430030 Hubei China

**Keywords:** SARS-CoV-2, COVID-19, CAR T-cell, Hematological malignancies, Treatment, Recommendations

## Abstract

The outbreak of coronavirus disease 2019 (COVID-19) posed an unprecedented challenge on public health systems. Despite the measures put in place to contain it, COVID-19 is likely to continue experiencing sporadic outbreaks for some time, and individuals will remain susceptible to recurrent infections. Chimeric antigen receptor (CAR)-T recipients are characterized by durable B-cell aplasia, hypogammaglobulinemia and loss of T-cell diversity, which lead to an increased proportion of severe/critical cases and a high mortality rate after COVID-19 infection. Thus, treatment decisions have become much more complex and require greater caution when considering CAR T-cell immunotherapy. Hence, we reviewed the current understanding of COVID-19 and reported clinical experience in the management of COVID-19 and CAR-T therapy. After a panel discussion, we proposed a rational procedure pertaining to CAR-T recipients with the aim of maximizing the benefit of CAR-T therapy in the post COVID-19 pandemic era.

## Introduction

The pandemic caused by severe acute respiratory syndrome coronavirus 2 (SARS-CoV-2) has posed extraordinary challenges for public health systems since December 2019 [1]. SARS-CoV-2 has developed gene mutations during its transmission, leading to the emergence of variants of concern, including Alpha, Beta, Gamma, Delta and Omicron with multiple subvariants and sub-branches [1, 2] - XBB being a trend as a dominant epidemic strain recently [3].Patients with hematological malignancies have higher risks of SARS-CoV-2 infection and more severe COVID-19 illness, ranging from 56.4 to 73.8% hospitalization rate, 9.8–24.1% intensive care unit (ICU) admission rate, 13.8–29.2% mechanical ventilation rate, and 31.2–62% mortality rate [4–6]. Although the World Health Organization (WHO) declared on May 5, 2023, that the pandemic no longer constitutes a ‘public health emergency of international concern’, COVID-19 is still present in localized small outbreaks [7]. The risk of breakthrough infections and severe outcomes, such as hospitalization and death, is still high for patients with hematological malignancies in the post-pandemic era [7]. Hence, how to manage this frail patient population is highly challenging.

CAR-T therapy is one of the major breakthroughs in the field of cancer immunotherapy and has revolutionized the prognosis of patients with malignancies [8, 9]. To date, six commercial CAR-T products targeting CD19 or B-cell maturation antigen (BCMA) have been approved by the FDA for the treatment of relapsed or refractory acute lymphoblastic leukemia, B-cell non-Hodgkin lymphoma (B-NHL) and multiple myeloma (MM) [10]. Two commercial products (Axi-Cel and Relma-Cel) targeting CD19 have been approved in China for the treatment of large B-cell lymphoma and follicular lymphoma (grade 3b) [11]. These patients achieved high remission rates and improved overall survival when CAR-T therapy was administered [10, 12]. In fact, with the widespread use of commercial CAR-T products, an increasing number of patients are receiving CAR-T therapy in the real world. Chinese scientists have made substantial contributions to improving the efficacy and safety of cellular immunotherapy [12–14]. China is now the country with the fastest development in the field of CAR T-cell research, with a rapid increase in the number of clinical trials since 2016, of which the number was largest in 2021 (108), followed by 2019 (101) and 2020 (99) [12–14]. A wide variety of clinical trials, such as those on dual-targeting CAR-T [15, 16], ‘off the shelf’ universal CAR-T [17, 18], TCR-T [19] and CAR-NK [20, 21], are ongoing. The number of innovative CAR-T products and the volume of investigational new drug applications involving CAR T-cells are also continually increasing, which corresponds to the increase in the number of clinical trials [22–27]. It is foreseen that more CAR-T products with different targets will be approved in the near future. However, CAR-T recipients face considerable complications and challenges amid the COVID-19 pandemic, with higher rates of severe/critical disease, longer hospitalization, and higher mortality [28–30]. Risk factors include attributes of the primary disease, its prior treatment, age, and comorbidities, among others [6, 31]. The highest mortality is observed in acute myeloid leukemia; the mortality rates of patients with B-NHL and MM are comparable [32]. CAR- T therapy itself is also an important risk factor. In BCMA-CAR-T patients, immunoglobulin deficiency is pronounced [33]. Humoral immunity is substantially impaired in patients who have received CD19-targeting CAR T cells, although somewhat long-lived plasma cells exist [34]. In general, patients are characterized by durable B-cell aplasia, hypogammaglobulinemia and a loss of the diversity of the T-cell repertoire after BCMA/CD19-targeted CAR-T therapy [35, 36]. The influence of different CAR-T targets on the immune system is summarized in Table 1. For the first time, we comprehensively reported the clinical manifestations and outcome of one BCMA CAR-T recipient with severe COVID-19 infection, emphasizing the importance of CAR-T recipient management during the COVID-19 pandemic [44]. Overall, responders to CAR-T salvage therapy experience considerable health challenges upon SARS-CoV-2 infection due to critical immune repertoire depletion [45]. In the post pandemic era, how to manage CAR-T recipients more safely and effectively is of the utmost importance. However, there is a lack of available guidelines and stringent clinical evidence to establish a standard procedure for treating COVID-19 in these fragile patients [45].

**Table 1:** The influence of different CAR-T targets on the immune system

Antigen	Expressed on which cells	Effect on the immune system	Tumor Target	The issue of COVID-19
**B-cell leukemia/lymphoma**				
CD19*	Most B-cell malignancies and most normal B-cell lineages	• B-cell aplasia contributes to hypogammaglobulinemia and impaired humoral immunity • Neutropenia	B-ALL, DLBCL, FL, MCL	• High risk of severe/critical SARS-CoV-2 [6, 29] • Longer hospitalization [6, 29] • High mortality rate [6, 29] • Low serological response rate after vaccination (57%) [37]; may have lower serological response rate compared with BCMA-based CAR-T (76.4%) [38] • Low rates of seroconversion and prolonged viral shedding [39, 40] • Longer duration of COVID-19 symptoms [29] • Prone to reinfection and rebound positivity • May be more likely to give rise to multimutational SARS-CoV-2 variants [41]
CD22	Most B-cell malignancies and most normal B-cell lineages		B-NHL	
CD20	Most B-cell malignancies and most normal B-cell lineages		B-ALL	
**Multiple myeloma**				
BCMA*	Mainly in plasma blast cells and terminally differentiated plasma cells	• B-cell aplasia contributes to hypogammaglobulinemia and impaired humoral immunity • Neutropenia	MM, PPCL, POEMS, AL	• As described above, the concern is that there may be a higher mortality rate (41%) [42] than with CD19 CAR-T (20%) [43]
GRPC5D	Highly expressed on normal and malignant plasma cells		MM	
**T-cell leukemia/lymphoma**				
CD7	T cells and natural killer cells and their precursors	• T-cell aplasia contributes to impaired cellular immunity • Neutropenia	T-ALL, T-LBL	• Healthy donor T cells may increase the risk of GVHD after COVID-19 • Delayed viral clearance
CD5	All T cells and B1 cell subset		T-ALL	
**Acute myelogenous leukemia**				
CD123	AML cells; B-ALL cells; HD; hematopoietic stem cells; dendritic cells	• Effect on hematopoiesis • Damage to hematopoietic stem cells	AML	• Delayed recovery of hematopoietic function
**Hodgkin lymphoma**				
CD30	Highly expressed on HD and anaplastic large-cell lymphoma; activated T cells, B cells and NK cells	• Affect the balance of the T-cell subsets • Transient neutropenia	HD	• Delayed viral clearance

* CAR-T cell products have been approved by the Food and Drug Administration and/or National Medical Products Administration

B-ALL: B-lineage acute lymphoblastic leukemia; DLBCL: diffuse large B-cell lymphoma; FL: follicular lymphoma; MCL: mantle cell lymphoma; B-NHL: B-lineage non-Hodgkin lymphoma; HD: Hodgkin’s lymphoma; T-ALL: T-lineage acute lymphoblastic leukemia; T-LBL: T-lineage lymphoblastic lymphoma; AML: acute myeloid leukemia; MM: multiple myeloma

With the profound understanding of COVID-19 and accumulation of experience in the management of CAR-T recipients, we summarized the *status quo* and provided management strategies for CAR-T recipients for use based on the latest domestic and worldwide COVID-19 prevention and treatment guidelines and the latest evidence-based medical knowledge, with the aim of improving the practical efficiency and benefit maximization of CAR-T therapy in the post-COVID-19 pandemic era [1, 46–48].

## Methods

Based on the National Regional Medical Center as well as Professional Committee for Evidence-based Medicine of the Chinese-German Society of Medicine (CEMOS), the discussion panel consisted of a multidisciplinary and interprofessional team, including hematologists, oncologists, respirologists, cardiologists, neurologists, infectiologists, and immunologists who were assembled to draft recommendations. Evidence was sourced from PubMed searches of original observations, studies, trials, meta-analyses, and key reviews. The disease severity of COVID-19 was assigned according to WHO classification criteria [49]. Specifically, the definition of critical COVID-19 was the same as the criteria for acute respiratory distress syndrome, sepsis, and septic shock [49]. In addition, any other conditions that would generally require the support of life-sustaining therapies such as mechanical ventilation (invasive or noninvasive) or vasopressor therapy could be defined as critical COVID-19 [49]. Severe COVID-19 was defined by any of the following conditions: oxygen saturation in room air less than 90% and signs of pneumonia or severe respiratory distress [49]. The lack of any criterion for severe/critical COVID-19 was defined as nonsevere COVID-19 [49]. A decision was made not to grade the level of evidence due to the lack of randomized controlled trials in CAR-T therapy. To provide more effective guidance for clinical practice, the strength of recommendations was categorized as ‘strong’ or ‘weak or conditional’ based on the available evidence, accessibility, and level of agreement, following the classifications set by the WHO guidelines for therapeutics and COVID-19 [49]. All panelists provided comments on the manuscript in multiple rounds until they reached an agreement. Lastly, a virtual finalizing seminar was conducted, during which the final version was reviewed and approved by all authors.

### Assessment and management of patients when preparing CAR-T therapy

**Fig. 1:**
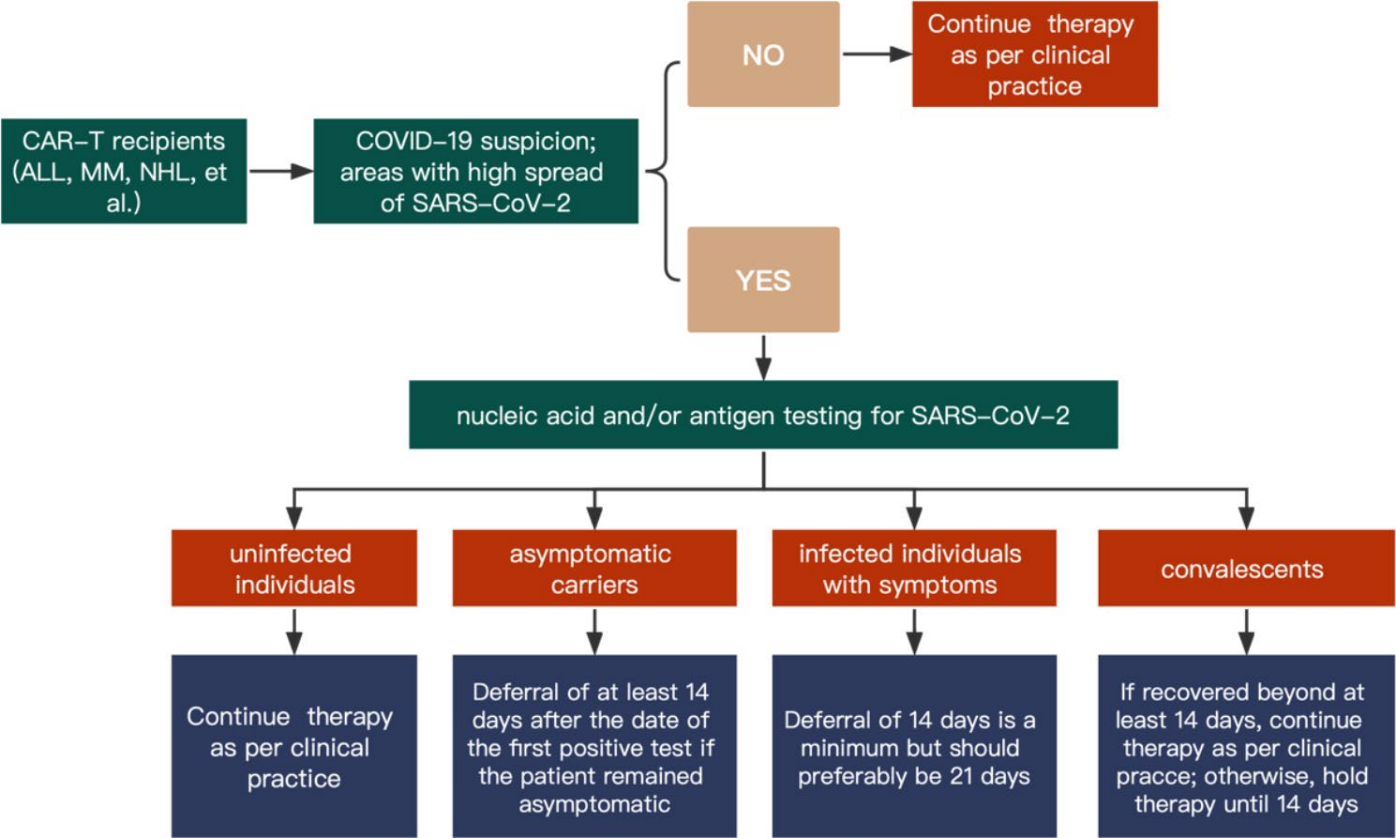
**The recommended timing of delayed therapy for patients with suspected COVID-19 when preparing for CAR-T therapy.** ALL: acute lymphoblastic leukemia; MM: multiple myeloma; NHL: non-Hodgkin lymphoma

Before proceeding with CAR-T therapy, it is necessary to fully assess the SARS-CoV-2 infection status. The investigation of epidemiological exposure histories and clinical COVID-19-related manifestations (i.e., pharyngalgia, cough, fever, myalgia/ arthralgia, headache, nasal discharge, etc.) is essential [50]. SARS-CoV-2 nucleic acid and/or antigen testing can further help in assessing infection status. Patients can be classified into three categories according to the status and timing of SARS-CoV-2 infection. CAR-T recipients are susceptible to infection with several respiratory viruses simultaneously. Screening for additional infections is necessary during the initial assessment (Table 2). Figure 1 shows the recommended timing of delayed CAR-T therapy in patients with suspected COVID-19.

**Table 2:** Assessment and screening of infection risk before CAR-T therapy

Tests	Contents	Patient status
**General laboratory tests**		
CBC, hepatic and renal function, coagulation function, etc.	Hb, WBC, PLT, ALT, AST, ALB, TBIL, LDH, UREA, CREA, BUN, PT, TT, APTT, etc.	For all patients.
**Inflammatory indicators** D-dimer, CRP, procalcitonin, serum ferritin, erythrocyte sedimentation rate, etc. Serum cytokines	IL-2, IL-4, IL-6, IL-10, IL-17, TNF-α, INF-γ, etc.	For SARS-CoV-2 confirmed patients to assess the degree of infection.
**Immune indicators** Lymphocyte subsets	total T lymphocytes (CD3^+^), helper/inducer T lymphocytes (CD3^+^CD4^+^), suppressor/cytotoxic T lymphocytes (CD3^+^CD8^+^), B lymphocytes (CD3^−^CD19^+^), and natural killer cells (CD3^−^CD16^+^CD56^+^), etc.	For all patients to evaluate their immune status.
**Etiological/serological detection**		
SARS-CoV-2	nucleic acid and/or antigen detection	For all patients and repeated if necessary.
HBV	HBV-DNA, HBsAg, HBsAb, etc.	For all patients.
Other respiratory viruses	nucleic acid, antigen, or antibody detection (including influenza A or B, RSV, parainfluenza viruses, adenovirus, enterovirus, rhinovirus, etc.)	When a respiratory tract co-infection is suspected. Note: It is difficult to distinguish COVID-19 from other respiratory virus infections based on symptoms alone.
Sputum cultures		The presence of sputum expectoration.
G/GM test		For patients with high-risk factors for fungal infection.
mNGS		Optional. An unbiased technology to detect a variety of pathogenic microorganisms.
bronchoalveolar lavage fluid through a bronchoscope	SARS-CoV-2 nucleic acid	Advisable if chest CT imaging suggests residual shadow.
**Imaging**		
Chest CT scans		For SARS-CoV-2 confirmed patients with pneumonia.
**Others**		If other infection or organ dysfunction is suspected, the appropriate examinations can be performed.

CBC, complete blood count; Hb, hemoglobin; WBC, white blood cell; PLT, platelet; ALT, alanine aminotransferase; AST, aspartate transaminase; ALB, albumin; TBIL, total bilirubin; LDH, lactate dehydrogenase; CREA, creatinine; UA, uric acid; BUN, blood urea nitrogen; PT, prothrombin time; TT, thrombin time; APTT, activated partial thromboplastin time; CRP, C-reactive protein; HBV, hepatitis B virus;HBsAg, hepatitis B surface antigen; HBsAb, hepatitis B surface antibody; RSV, respiratory syncytial virus; G/GM test, 1,3-β-D glucan test/galactomannan antigen test; mNGS, metagenomic next-generation sequencing

### 1. CAR-T recipients without SARS-CoV-2 infection

This category of patients is defined as negative in SARS-CoV-2 nucleic acid/antigen testing without clinical manifestations. The general treatment protocol for those patients can adhere to the standard CAR-T procedure [51, 52]. However, these patients are at high risk of contracting SARS-CoV-2 and of having a severe disease course during CAR-T therapy in local COVID-19 epidemic areas [45, 53]. It is crucial for these patients to avoid infection by wearing adequate personal protective equipment and undergoing testing and surveillance for SARS-CoV-2, particularly in local high-transmission areas [54].

### Recommendations

- Nonpharmaceutical interventions are still needed, including social distancing, reinforced hygiene, and the use of individual protection devices such as face masks, face shields, and other types of personal protective equipment [55, 56]. (Strong recommendation)
- In areas with localized outbreaks of COVID-19, single-ward living, and laminar-flow beds are recommended during the peri-CAR-T period. (Strong recommendation)
- SARS-CoV-2 infection status must be re-evaluated at three important time points: prior to leukapheresis, prior to lymphodepleting conditioning (LD), and prior to CAR T-cell infusion [57, 58]. (Strong recommendation)

### 2. CAR-T recipients infected with SARS-CoV-2

CAR-T therapy should be delayed or suspended if patients are confirmed to have SARS-CoV-2 infection. McNerney et al. reported that half of patients infected with SARS-CoV-2 before CAR-T had their CAR T-cell infusion delayed by 15 ~ 30 days [59]. However, patients who defer CAR-T therapy are at high risk for the progression of the underlying disease [60]. Therefore, a multidisciplinary team (MDT) is needed to provide supportive treatment, fully balancing the risks of delayed CAR-T therapy and SARS-CoV-2 infection. The European Society for Blood and Marrow Transplantation (EBMT)-Joint Accreditation Committee of the ISCT and EBMT (JACIE) and the European Hematology Association (EHA) recommend that asymptomatic patients who test positive for SARS-CoV-2 nucleic acid may proceed to CAR-T manufacture [31]. However, subsequent therapy, including LD and CAR T-cell infusion, should be delayed/suspended due to the risk of infection aggravation. Nonetheless, delayed CAR-T therapy can be appropriately shorter than that in symptomatic patients if they experience rapid disease progression [61]. No guideline is available on whether to initiate antiviral therapy in this case.

## Recommendations

- Whenever SARS-CoV-2 infection occurs (before/after leukapheresis and LD), subsequent treatment should be suspended until the patient becomes asymptomatic and has two negative virus nucleic acid swabs at least 24 h apart. A suspension of 14 days is a minimum duration but should preferably be 21 days, depending on the severity of COVID-19 (i.e., nonsevere, severe, and critical disease) [49, 62]. The suspension can be appropriately extended if the primary condition remains relatively constant to minimize the risk associated with CAR-T therapy. (Strong recommendation)
- If chest CT imaging suggests a residual shadow, SARS-CoV-2 nucleic acid detection in bronchoalveolar lavage fluid is necessary to re-evaluate the status of COVID-19 [63, 64]. (Strong recommendation)
- In patients who are negative for SARS-CoV-2 nucleic acid/antigen but still have respiratory signs and symptoms, further assessment of pulmonary function is needed to determine whether the patient is suitable for CAR-T therapy initiation. (Strong recommendation)
- For asymptomatic carriers, leukapheresis can be performed, and CAR T-cells can be manufactured. LD and CAR T-cell infusion should be held/suspended for at least 14 days after the date of the first positive test if the patient remains asymptomatic [61]. (Strong recommendation)
- If a patient has persistently positive nucleic acid amplification tests beyond 30 days, additional testing could include digital droplet PCR (polymerase chain reaction), viral genome sequencing to surveil the emergence of new variants of SARS-CoV-2 or attempts to identify replication of the competent virus in conjunction with infectious disease consultation [61]. (Weak or conditional recommendation)

### 3. CAR-T recipients who have recovered from SARS-CoV-2 infection

This category of patients is defined as having recovered clinical manifestations, significant absorption of pulmonary chest lesions on CT imaging, and two consecutive negative SARS-CoV-2 tests from a respiratory specimen (at least 24 h between samples) [65]. However, CAR-T recipients usually have an impaired humoral response and low seroconversion rates following SARS-CoV-2 infection, especially those pretreated with rituximab and multiline chemotherapies [6]. Hence, reinfection remains a concern for these patients.

### Recommendations

- For patients who have been infection free for at least 14 days, CAR-T therapy can adhere to the standard CAR-T procedure. Otherwise, therapy is typically suspended until 14 days have passed [51]. For patients who have recovered from severe/critical SARS-CoV-2 infection, a suspension of 21 days or longer is preferable, depending on the progression of the primary disease [52, 66]. (Strong recommendation)
- Caution against reinfection should still be taken during the process of CAR-T therapy [67, 68]. Personal protection is still necessary. Regular SARS-CoV-2 nucleic acid/antigen testing is recommended. (Strong recommendation)

### Management and treatment strategies for COVID-19 patients post CAR T-cell infusion

CAR-T recipients are susceptible to SARS-CoV-2 infection and are at higher risk for COVID-19 complications, including hospitalization, ICU admission, mechanical ventilation, and death [6]. According to a report of 57 patients diagnosed with COVID-19 after CAR-T therapy from 11 countries, 80% of patients had to be admitted to the hospital for COVID-19-related symptoms, 42.9% needed oxygen support, and 39.3% were admitted to the ICU; the COVID-19-attributable mortality rate was 41.1%, which was mostly 10 ~ 40 times higher than that reported in the general population [29]. Overall, the management of these patients is highly challenging and difficult. In SARS-CoV-2 epidemic areas, it is recommended to perform CAR-T treatment in more established CAR-T medical facilities to achieve optimal whole-course management, and the involvement of an MDT is important. According to the temporal proximity of SARS-CoV-2 infection after CAR T-cell infusion, the timing can be divided into three categories: short-term (0–28 days), medium-term (28–100 days) and long-term (> 100 days) [46]. SARS-CoV-2 can be present at multiple anatomical sites and persist for several months in the body, especially in immunocompromised patients [69, 70]. For CAR-T recipients, particularly those treated with BCMA targeting therapies, persistent B-cell aplasia, lymphopenia and hypogammaglobulinemia can lead to delayed virus clearance post CAR T-cell infusion [44, 71−73]. However, prolonged viral shedding (mean of 61 days) may lead to genomic evolution of the virus with the emergence of new variants of concern as defined by the WHO, bringing more difficult challenges for treatment, and even breaking through existing immune barriers, leading to new epidemics [6, 41, 74]. The patient’s immune status is influenced not only by the primary disease but also by the product phenotype, composition, and dose of CAR-T cells [75]. We believe that CAR-T recipients’ immune status is critical for the treatment response and outcome of COVID-19, especially in severe/critical patients. Herein, the recommendations for different terms are not constant. Dynamic assessment of immune status, including lymphocyte absolute value and function, is important, and COVID-19 therapy needs to be individualized.

Once patients are confirmed to have SARS-CoV-2 infection, active treatment should be initiated. The average viral load in severe cases was found to be approximately 60 times higher than that in nonsevere cases, suggesting that higher viral loads may be associated with severe clinical outcomes [76]. Early virus clearance was found in patients with nonsevere cases, and 90% of these patients tested negative by repeated PCR on day 10 after onset [76]. In contrast, all patients with severe/critical disease continued to test positive on or after the 10th day of onset [76]. Early and prompt antiviral treatment can substantially reduce the rate, quantity, and duration of viral replication and the viral load and accelerate clearance of the virus, contributing to improved symptoms, delayed disease progression, and reduced mortality [77–79]. The time window from illness onset to antiviral therapy is significantly correlated with the duration of negative nucleic acid test results [78]. Therefore, regardless of the period of infection, active antiviral treatment is the cornerstone. Currently, antiviral agents, such as paxlovid, molnupiravir, remdesivir, and azvudine, are available, with paxlovid being preferred [80–83]. Notably, paxlovid is a P450 3A4 inhibitor, inducer of 2C9 and 2C19, and P glycoprotein inhibitor that has multiple drug interactions with a wide range of drugs; therefore, drug interaction screening must be performed when it is administered [84, 85]. Moreover, hepatic, and renal toxicity is another concern with most antiviral drugs [86–89]. However, molnupiravir is less prone to drug interactions and no dose adjustment is required for renal or hepatic impairment [90]. The PCR cycle threshold value for SARS-CoV-2 detection can reflect the relative viral load. Monitoring the dynamic change in the cycle threshold value can partially evaluate antiviral effectiveness [91]. High titers of COVID-19 convalescent plasma, which is preferably obtained from those who have recovered from the omicron variant and have been previously vaccinated, may be beneficial for immunocompromised patients with persistent SARS-CoV-2 infection, especially those with B-cell impairment [61, 92, 93]. Effective monoclonal antibody therapy depends upon the current and predominant SARS-CoV-2 variant, which may rapidly change, and the neutralizing activity is constantly challenged by new variants [94]. Human COVID-19 immunoglobulin can be administered to patients with a high viral load and rapid disease progression [95]. Although intravenous immunoglobulins (IVIGs) are unable to elicit SARS-CoV-2-neutralizing antibodies, replacement is strongly recommended to complement immune deficiency in CAR-T recipients [31].

CAR-T recipients are also at high risk of infections with various other pathogens. Most early infections (first 28 days) are bacterial or respiratory viral infections. Invasive fungal infections are rare during this period [31]. Beyond day + 28, viral infections predominate, and opportunistic infections are common [31]. Thus, more attention needs to be paid to COVID-19 CAR-T recipients complicated with other pathogen infections. Timely initiation of appropriate therapy is important to minimize the risk of mortality.

Another issue is that the causes of cytokine release syndrome (CRS) or the cytokine storm are difficult to identify in CAR-T recipients infected with SARS-CoV-2. Both CAR-T therapy and COVID-19 can cause CRS. CRS affects 30%~100% of CAR-T recipients [31]. Profiling serum cytokine levels reveals that IL-6 and IL-10 are disease severity predictors in COVID-19 patients [96]. Moreover, encephalopathy related to the cytokine storm is present in a subgroup of COVID-19 patients and has several overlapping features with immune effector cell-associated neurotoxicity syndrome (ICANS) caused by CRS, including headache, impaired cognition and attention, dysexecutive syndrome, language disturbances, akinetic mutism, delirium, and transitory motor deficits [97]. SARS-CoV-2 can rapidly provoke immune responses by pathogenic Th1 cells, monocytes, macrophages, and dendritic cells to produce large quantities of proinflammatory cytokines, including IL-6 and TNF-α, thus leading to the occurrence of CRS [98, 99]. Activation of the angiotensin 2 pathway is another potential mechanism involved in the cytokine storm in COVID-19 patients [100]. Moreover, sepsis can similarly trigger the release of inflammatory cytokines [101]. In summary, identification of the trigger of CRS is difficult. Regardless of the cause, early intervention with anti-inflammatory therapy is necessary. The IL-6 receptor inhibitor tocilizumab is approved and widely used to treat CRS post CAR T-cell infusion [58, 60]. However, in COVID-19 CAR-T recipients, systemic corticosteroids are the first choice for alleviating any CRS, although they may affect the efficacy of CAR-T therapy [63, 102]. Tocilizumab and JAK inhibitors can be considered if CRS cannot be effectively controlled [103]. Among the FDA-approved JAK inhibitors, baricitinib has the most data from evidence-based medicine for severe/critical COVID-19 patients, although ruxolitinib [104–107] is used more broadly for hematological patients. Herein, we recommend baricitinib as the first-choice JAK inhibitor if needed. Notably, the use of systemic corticosteroids, IL-6 inhibitors, and JAK inhibitors can further increase the risk of infection [108, 109].

Regarding allogeneic CAR-T therapy, donors are required to be negative for SARS-CoV-2 nucleic acid/antigen [52]. In addition, SARS-CoV-2 infection may induce acute graft-versus-host disease (aGVHD) or exacerbate GVHD in patients with a history of aGVHD, including liver, gastrointestinal tract, and skin GVHD [48]. If this occurs, treatment for GVHD is needed at the same time as antiviral therapy, and the principle of this treatment is generally the same as that post allogeneic stem cell transplantation [110]. Systemic corticosteroids are still the preferred initial treatment for aGVHD [111]. However, given the risk of aggravated infection with corticosteroids, mesenchymal cell infusion can be used earlier to treat both aGVHD and COVID-19-induced CRS [112].

**Fig. 2:**
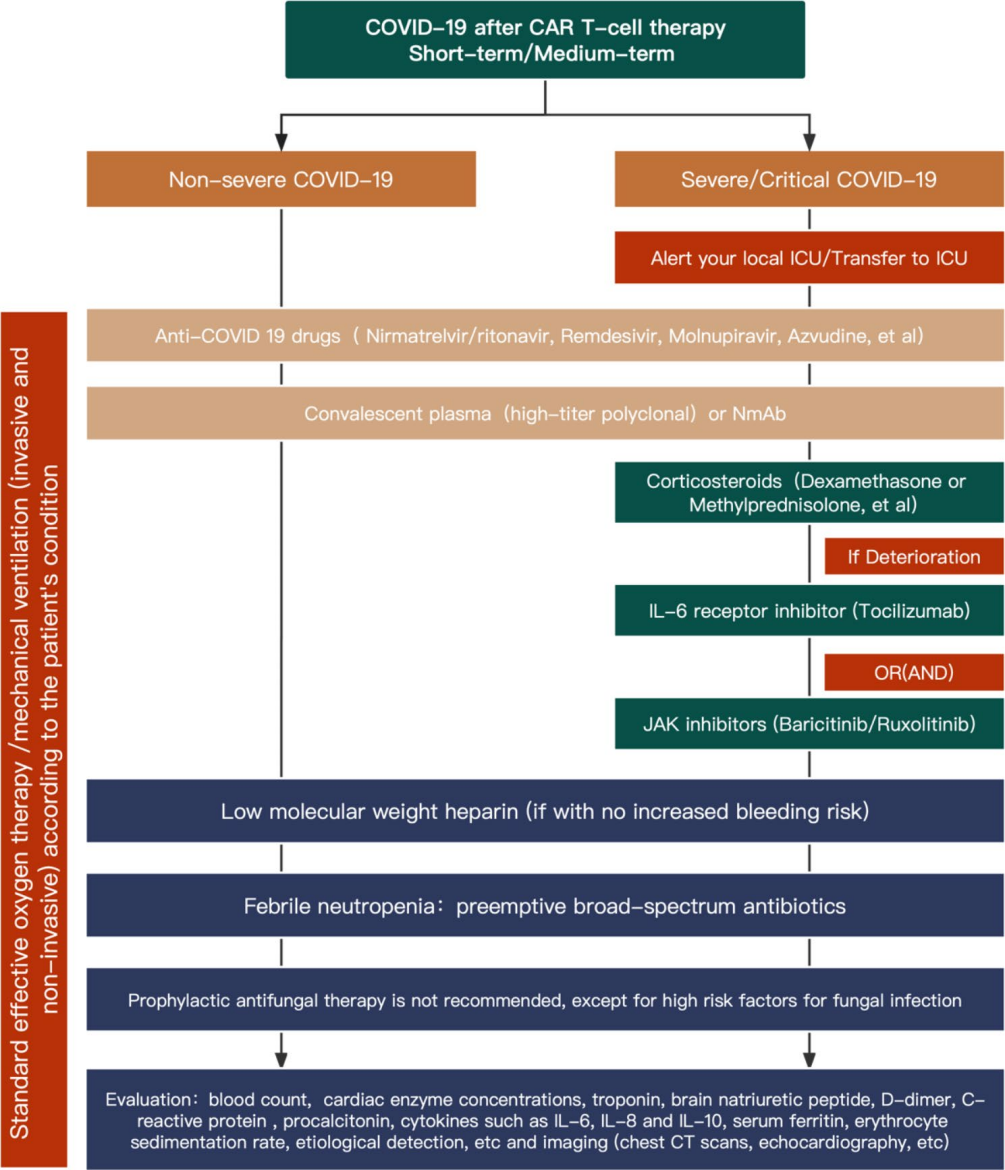
**Management of COVID-19 patients in the short/medium term (< Day + 100) post CAR T-cell infusion. **ICU, Intensive care unit; NmAb, neutralizing monoclonal antibodies

**Fig. 3:**
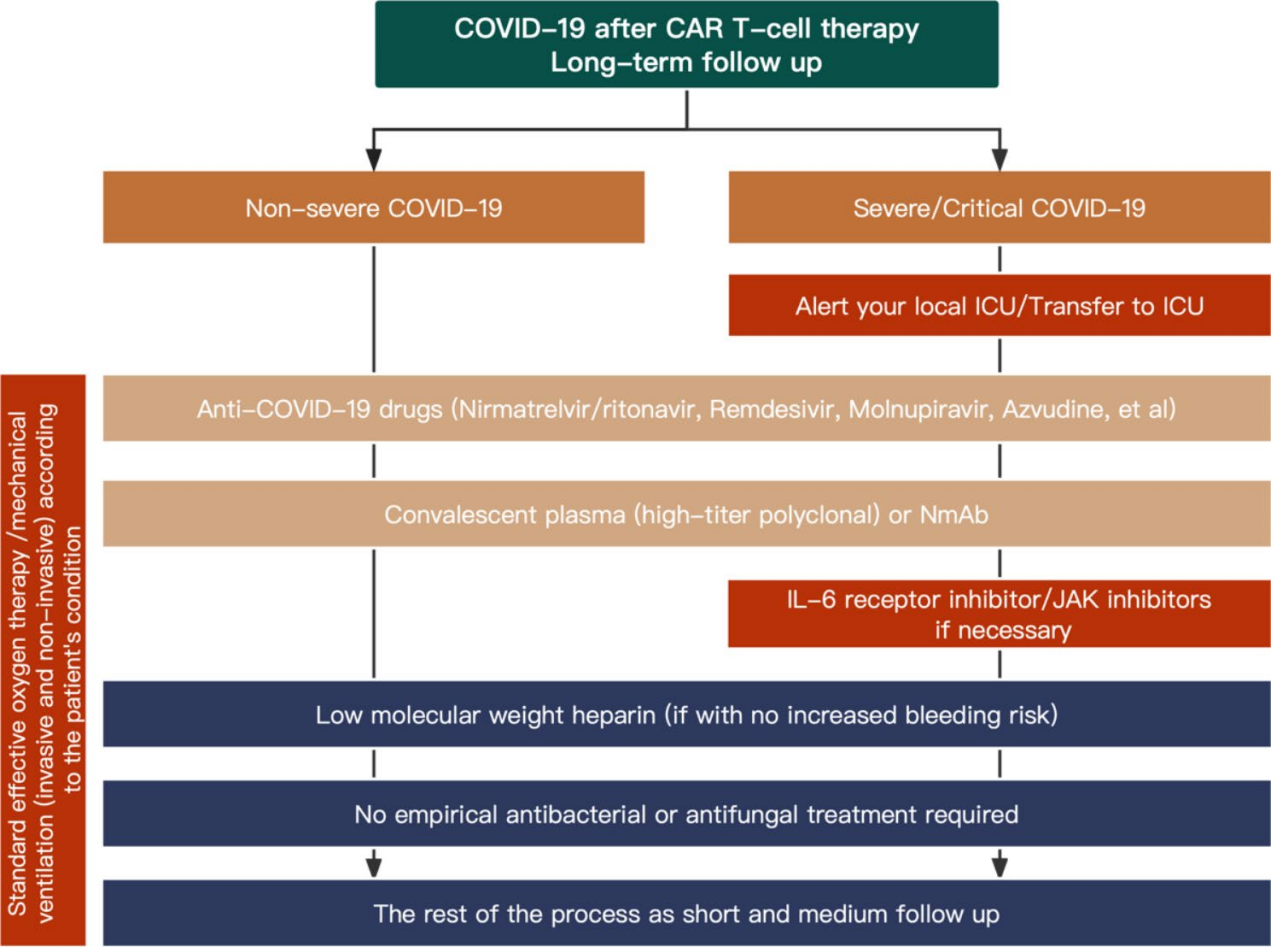
**Management of COVID-19 patients in the long-term (> Day + 100) post CAR T-cell infusion.** ICU, Intensive care unit; NmAb, neutralizing monoclonal antibodies

The management of COVID-19 patients in the short/medium term and long-term after CAR T-cell infusion is summarized in Figs. 2 and 3, respectively. The treatment principles for CAR-T recipients infected with SARS-CoV-2 include (1) general treatment, ensuring adequate calorie and nutrition intake and maintaining water/electrolyte balance and homeostasis; (2) standard effective oxygen therapy measures given according to the severity of COVID-19, including a nasal catheter or mask oxygen therapy and high-flow nasal oxygen inhalation; (3) early and prompt antiviral treatment; (4) immunomodulatory and anticoagulant therapy and supportive regimens given according to the severity of COVID-19; (5) coinfection with other respiratory pathogens, such as influenza viruses, parainfluenza viruses, adenovirus, and respiratory syncytial virus, should be noted, for which oseltamivir is recommended [113–115]; (6) attention should be given to drug-drug interactions and drug toxicity; (7) passive immunotherapy, such as high-titer convalescent plasma, monoclonal antibodies and human COVID-19 immunoglobulin, may be beneficial [116–118]; and (8) IVIGs should be administered for hypogammaglobulinemia (IgG < 4 g/l) [31]. Recommendations for treatment agents are summarized in Table 3.

**Table 3:** Overview of drugs and recommendations for COVID-19 CAR-T recipients

Drugs	Key issues to consider	Strength of recommendation
**Antiviral drugs**	The duration and course of antiviral treatment can be appropriately prolonged.	
Nirmatrelvir and ritonavir (Paxlovid) (Target: 3CL)	It is essential to monitor for drug-drug interactions, and the dosage should be modified in accordance with renal function. The COVID-19 drug interactions query website is https://covid19-druginteractions.org/checker.	Strong
Remdesivir* (Target: RdRp)	Remdesivir should not be taken if ALT > 10 ×ULN or if ALT levels are increased and there are symptoms of active hepatitis.	Weak or conditional
Molnupiravir (Target: RdRp)	No dose adjustment is required for renal or hepatic impairment.	Weak or conditional
Azvudine (Target: RdRp)	It is not recommended to use during pregnancy or lactation. Patients with moderately to severely impaired liver and kidney function should use it with caution.	Weak or conditional
Monoclonal antibodies	Patients with major risk factors for disease progression, high viral loads, and rapid disease development (depending upon predominant circulating viral variants).	
Bebtelovimab*	It is effective against all Omicron subvariant virus strains (including BA.1, BA.1.1, and BA.2).	Weak or conditional
Tixagevimab/cilgavimab*	It is only recommended for preexposure prophylaxis.	Weak or conditional
Convalescent plasma	The patient’s individual condition and viral load should be considered while determining whether to administer again.	Weak or conditional
Human COVID-19 immunoglobulin	Patients with major risk factors for disease progression, high viral loads, and rapid disease development. According to the patient’s condition, it can be infused once again the next day; the total number of infusions should be no more than 5.	Weak or conditional
**Immunoregulatory drugs**		
Corticosteroids	Patients with critical and severe conditions that display rapid imaging progression, a body inflammatory response that is aggressive, and a steadily declining oxygenation index. The early use of systemic corticosteroids for severe and critical patients is emphasized. Patients with severe CRS could benefit from high-dose corticosteroid therapy.	
Dexamethasone	The dosage of dexamethasone should be adjusted to the severity of CRS. The dosage can be appropriately increased to 10 mg/6 h for 1–3 days in patients with ICANS.	Strong
Methylprednisolone	If ICANS symptoms are still not relieved following the use of dexamethasone, methylprednisolone may be administered. The dosage of methylprednisolone is 1000 mg/day for 3 days, 250 mg × 2/day for 2 days, 125 mg × 2/day for 2 days, and 60 mg × 2/day for 2 days.	Strong
**IL-6 inhibitors**		
Tocilizumab	If CRS is exacerbated, combination therapy with IL-6 inhibitor tocilizumab (8 mg/kg) is recommended. Tocilizumab should be used with caution in the case of ICANS.	Strong
**JAK inhibitors**		
Baricitinib	Attention should be given to symptoms and warning indications of thromboembolic events, and coagulation indicators should be identified when necessary.	Strong
Ruxolitinib	Most clinical criteria, symptoms (such as respiratory distress or the need for oxygen), and other clinical indications are used to determine whether to begin the use of ruxolitinib.	Weak or conditional
**IVIGs**	It is recommended for hypogammaglobulinemia (IgG < 4 g/l).	Strong

* Not yet authorized for listing in China

ALT, alanine aminotransferase; ULN: upper limit of normal value; CRS, cytokine release syndrome; ICANS, immune effector cell-associated neurotoxicity syndrome; IVIGs, intravenous immunoglobulins

### 1. Short-term infection (< 28 days)

During this period, CAR-T recipients are prone to multiple complications such as CRS and infection, and the risk of disease exacerbation is high [46]. Following LD, all patients are neutropenic and have severe cellular immune deficiency, resulting in delayed clearance of SARS-CoV-2 [119, 120]. A high viral load is independently associated with increased mortality [121]. Therefore, blocking viral replication must be a priority to reduce mortality. Whether G-CSF and its derivatives applied earlier after CAR-T therapy increase the risk of CRS or ICANS has not been clearly demonstrated [46]. Moreover, empiric antimicrobial therapy is essential since neutropenia with fever more often occurs in this period. According to the findings of our previous study, invasive fungal infections were more frequently detected in patients with grade 3~5 CRS, among whom the probability was 23.08%; however, the probability was only 5.26% in the mild CRS group; thus, antifungal therapy can be considered if a fungal infection is confirmed [122]. During this period, there is a great overlap between the clinical symptoms caused by CAR-T therapy and those caused by SARS-CoV-2 infection, such as fever, CRS, and ICANS, meaning that differential diagnosis is often difficult. Regardless of the cause of CRS, active treatment is necessary, and laboratory examinations/evaluation intervals should be short, especially for grade 3 ~ 5 CRS, given the high risk of death in this period. Overall, standard therapy principles are lacking for this group of patients.

### Recommendations

- Once SARS-CoV-2 infection is confirmed, antiviral therapy must be initiated immediately. Oral antiviral agents, such as paxlovid, remdesivir, and azvudine, are recommended. The choice will depend on the availability of the abovementioned drugs. The duration of antiviral treatment can be appropriately prolonged, which depends on the timing of SARS-CoV-2 nucleic acid/antigen negativity and the patient’s clinical symptoms and immune status. (Strong recommendation)
- If neutropenia with fever occurs, empirical antimicrobial therapy is strongly recommended. Carbapenems and antibiotics containing enzyme inhibitors are preferred [123, 124]. (Strong recommendation)
- Prophylactic antifungal therapy is not recommended, except for those with grade ≥ 3 CRS, severe/critical SARS-CoV-2, or other high-risk factors for fungal infection [31]. (Strong recommendation)
- The application of granulocyte colony-stimulating factor (G-CSF), *granulocyte*-macrophage CSF, and polyethylene glycol G-CSF is controversial for neutropenia. However, they should still be avoided if CRS occurs. (Weak or conditional recommendation)
- If CRS occurs, systemic corticosteroids should be used in a timely manner. If CRS is exacerbated, it should be combined with tocilizumab. If CRS still cannot be effectively controlled, it should be combined with or adjusted to the JAK inhibitors baricitinib/ruxolitinib. The oxygen saturation and vital signs of patients should be closely monitored. Laboratory tests, such as those for cytokines, C-reactive protein, and ferritin, should be repeated every 12–24 h. (Weak or conditional recommendation)
- The principles of treatment for COVID-19-related encephalopathy are consistent with those for ICANS. The ICE score and CARTOX-10 score should be evaluated to guide treatment [125, 126]. Cerebrospinal fluid examination (cell counts, protein levels, inflammatory biomarkers, SARS-CoV-2 nucleic acid, etc.), electroencephalogram and brain MRI are important for differential diagnosis and to exclude alternative diagnoses. Other functional and symptom scales can be evaluated if necessary. (Strong recommendation)
- Critical patients should be monitored in the hematological ICU or ICU staffed with hematologists. Continuous renal replacement therapy could be considered to eliminate inflammatory cytokines and pathogens [127, 128]. Possible adverse events should be considered with caution. (Weak or conditional recommendation)

### 2. Medium-term infection (day + 28 to day + 100)

During this period, B-cell aplasia/hypogammaglobulinemia, prolonged neutropenia and CD4 T-cell lymphopenia contribute to immunodeficiency [31, 129, 130]. Due to their limited virus-specific humoral response and delayed viral clearance, COVID-19 CAR-T recipients are still at risk of developing severe disease, high hospitalization rates, and high mortality. Some patients will experience delayed CRS [31, 131, 132]. Supportive therapy is important, especially immunoglobulin replacement.

### Recommendations

- Once SARS-CoV-2 infection is confirmed, antiviral treatment should be started as soon as possible. (Strong recommendation)
- If pneumonia occurs, systemic glucocorticoids should be administered, and the doses and courses of the treatment should be adjusted according to the disease severity. The early use of systemic corticosteroids is critical for severe/critical patients. (Strong recommendation)
- Immunoglobulin replacement should be administered in patients with hypogammaglobulinemia (IgG < 4 g/l) [31]. (Strong recommendation)
- Intravenous human COVID-19 immunoglobulin and convalescent plasma may be given to patients with a high viral load and rapid disease progression during the early stage [95, 133–135]. (Weak or conditional recommendation)
- If patients have recurrent fever ≥ 38.0 °C, hemodynamic instability or hypoxemia, clinicians should be alert to the possibility of delayed CRS [31, 131, 132].

### 3. Long-term infection (> day + 100)

During this period, the impaired immunity of CAR-T recipients recovers to a certain degree [136]. However, the duration of virus clearance is still longer than that in healthy individuals. Active antiviral therapy is necessary. Patients are at relatively low risk of concurrent bacterial infections; hence, empiric antimicrobial therapy should be avoided. In addition, combination therapy, such as that with immune checkpoint inhibitors PD-1/PD-L1 monoclonal antibodies, immunomodulator lenalidomide, and targeted agents BTK inhibitors, may be administered in some cases after CAR T-cell infusion. The application of these drugs should be considered comprehensively during the period of SARS-CoV-2 infection.

### Recommendations

- The use of CD20 monoclonal antibodies such as rituximab and obinutuzumab should be suspended during acute episodes of SARS-CoV-2 infection. (Strong recommendation)
- The use of other agents, such as PD-1/PD-L1 inhibitors, BTK inhibitors and lenalidomide, remains controversial in COVID-19-confirmed CAR-T recipients. (Weak or conditional recommendation)
- For nonsevere COVID-19 patients, early antiviral intervention is still needed. Monitoring the levels of cytokines (IL-2, IL-4, IL-6, IL-10, IL-17, TNF-α, INF-γ, etc.), C-reactive protein, procalcitonin, and other inflammatory indicators is recommended. If disease exacerbation occurs, systemic corticosteroids, IL-6 and/or JAK inhibitors can be used, as appropriate. (Strong recommendation)
- For severe/critical patients with SARS-CoV-2, active immunomodulatory therapy is needed. (Strong recommendation)
- The inappropriate use of antibiotics, especially the use of broad-spectrum antimicrobial agents, should be avoided. (Strong recommendation)

### The issue of SARS-CoV-2 vaccination for CAR-T recipients

**Fig. 4:**
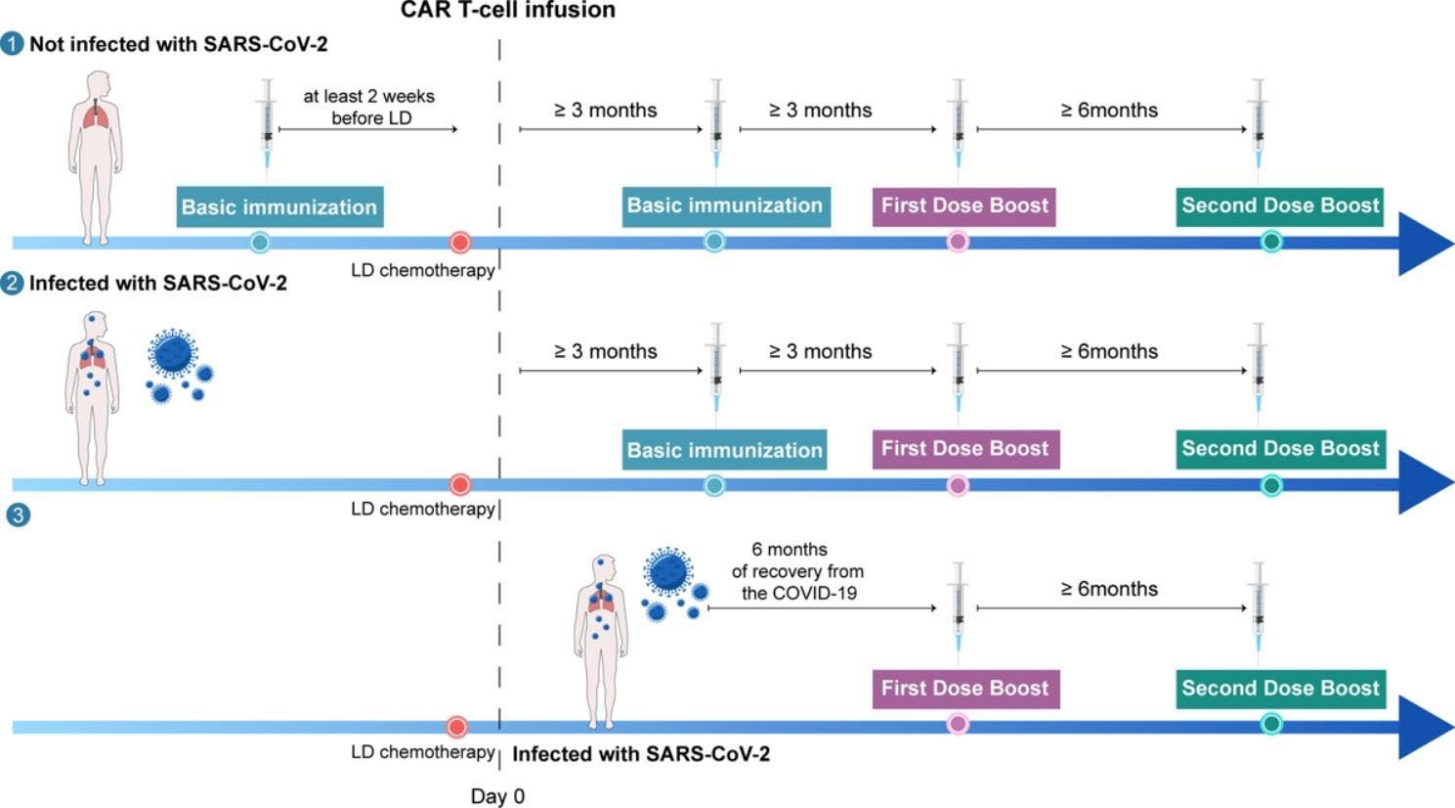
**SARS-CoV-2 vaccination for CAR-T recipients.** For individuals without infection, the initial vaccination series should be completed at least 2 weeks prior to beginning lymphodepleting conditioning. Regardless of infection or vaccination history, patients are advised to receive primary and booster vaccines after CAR-T treatment. If patients are infected with SARS-CoV-2 post CAR T-cell infusion, revaccination should be carried out 6 months after recovery from COVID-19. LD: Lymphodepleting conditioning

It’s known that vaccines are helpful to prevent hospitalizations and deaths after SARS-CoV-2 infection for all variants known to date [40, 137, 138]. While the antibody response in hematological patients following full SARS-CoV-2 vaccination may be lower compared to the general population, a clinical benefit is still observed [139–141]. Hence, COVID-19 vaccination is particularly recommended for CAR-T recipients before CAR-T therapy in the absence of contraindications [6, 142]. However, CAR-T therapy that targets B cells inevitably eliminates the immunological memory of the vaccine because it removes memory T cells and leads to B-cell lymphoproliferative disorders [143]. Thus, it is necessary to administer primary vaccines to these patients after CAR-T treatment, regardless of the history of previous infection or vaccination. Serological response rates varied significantly among patients treated with different CAR-T products, from 73.2% for BCMA or CD138-directed CAR-T recipients to only 22.8% for CD19-directed CAR-T recipients [39]. A meta-analysis shows that the serological response rate of CAR-T recipients receiving one dose of COVID-19 vaccine is 20.4% [144]. The serological response rate increased as the number of vaccinations increased among CAR-T recipients to 55.5% after 2 doses versus 62% after 3 doses and versus 75% after 4 doses [145]. The anti-S IgG titers after 4 doses of vaccine were more than 30 times higher than those after 2 doses [145]. Thus, the administration of booster vaccination is beneficial. Figure 4 shows the recommended timing of vaccination for CAR-T recipients.

### Recommendations

- Patients can be vaccinated with any of the available vaccines, including mRNA vaccines, adenoviral vector-based and inactivated SARS-CoV-2 vaccines, except live attenuated vaccines with replicating virus [146–149]. (Strong recommendation)
- Patients who plan to receive CAR-T therapy should complete their initial SARS-CoV-2 vaccination series at least 2 weeks prior to beginning LD depending on the status of the primary disease [46]. (Strong recommendation)
- The COVID-19 vaccine can be administered after 6 months of CAR-T therapy and as early as 3 months after CAR T-cell infusion in cases of high community transmission rates, at which point immune function gradually recovers and elicits vaccine-induced serological responses [149, 150]. (Strong recommendation)
- Three months after basic immunization, one booster dose is feasible, and the second dose may be considered 6 months later to obtain high antibody titers and a long period of protection [151]. (Strong recommendation)
- Booster vaccination can be performed using homologous or different types of vaccines, such as a homologous inactivated vaccine booster, heterologous inactivated vaccine and subunit vaccine booster, or heterologous mRNA vaccine and adenovirus vaccine booster [137].
- Patients can be revaccinated six months after they have recovered from COVID-19. (Weak or conditional recommendation)
- For unvaccinated individuals, individuals who postpone vaccination and individuals who are at high risk for COVID-19 but unable to mount an adequate immune response following vaccination, long-acting neutralizing antibodies for preexposure prophylaxis can be considered [152, 153]. (Weak or conditional recommendation)
- Health providers, caregivers and household partners should receive the initial immunization and complete booster immunization as early as possible. (Strong recommendation)

### Conclusion and future challenges

SARS-CoV-2 is still life threatening for CAR-T recipients, whose management is difficult and highly specialized. In countries or local communities where SARS-CoV-2 infection is widespread, continuous surveillance for early diagnosis of COVID-19 is essential. An MDT is needed to balance the risk of a potentially lethal infection and aggressive disease relapses if CAR-T therapy is interrupted due to COVID-19. If COVID-19 occurs, preemptive treatment based on early administration of antiviral drugs is crucial to limit the risk of developing severe COVID-19. Patients should choose more established CAR-T centers with an ICU for CAR-T treatment to receive better whole-course management. Preventive strategies remain the most important measures. Among them, vaccination should be promoted based on T-cell-driven immunization. New therapeutics, including CAR-T/CAR-NK therapies targeting SARS-CoV-2 antigens and ACE2-biospecific antibodies, are promising potential strategies for the treatment and prevention of COVID-19, despite their high treatment costs [154, 155]. We will probably experience occasional local COVID-19 epidemics over a certain period. CAR-T recipients need to be more vigilant about breakthrough infections. Thus, this work has not only important practical implications now but also plays an exemplary role on other potential pandemics, such as those caused by coronaviruses and other respiratory viruses which can cause severe pneumonia in the future. Furthermore, our research remains highly relevant for patients undergoing other cellular immunotherapies, such as CAR-NK and TCR-T cell [156, 157]. Notably, these multidisciplinary recommendations are based on currently available evidence and will need to be revised frequently with continuously accumulating randomized controlled trial data to provide high-quality evidence-based guidance.

## Data Availability

Not applicable.

## Abbreviations

aGVHD: Acute graft-versus-host disease
B-NHL: B-cell non-Hodgkin lymphoma
CAR: Chimeric antigen receptor
CEMOS: Professional Committee for Evidence-based Medicine of the Chinese-German Society of Medicine
COVID-19: Coronavirus disease 2019
CRS: Cytokine release syndrome
CSF: Colony-stimulating factor
ICANS: Immune effector cell-associated neurotoxicity syndrome
ICU: Intensive care unit
IVIGs: Intravenous immunoglobulins
LD: Lymphodepleting conditioning
MDT: Multidisciplinary team
MM: multiple myeloma
PCR: Polymerase chain reaction
SARS-CoV-2: Severe acute respiratory syndrome coronavirus-2
WHO: World Health Organization
